# Treatment of borderline hip dysplasia with triple pelvic osteotomy: preoperative values of acetabular index and lateral center edge angle can indicate overcorrection

**DOI:** 10.1007/s00402-023-04920-z

**Published:** 2023-06-05

**Authors:** Daniel Dornacher, Bernd Lutz, Michael Fuchs, Timo Zippelius, Heiko Reichel

**Affiliations:** Department of Orthopedics, University of Ulm, Oberer Eselsberg 45, 89081 Ulm, Germany

**Keywords:** Borderline hip dysplasia, Acetabular overcorrection, Triple pelvic osteotomy, Hip preservation surgery

## Abstract

**Introduction:**

After pelvic osteotomy for the treatment of symptomatic hip dysplasia, the longevity of the hip joint can be compromised by acetabular overcorrection. This iatrogenic pincer-type deformity is considered to be one of the major risk factors for persistent pain and progressing osteoarthritis. There is evidence that acetabula in the borderline range, defined by a lateral center edge angle (LCEA) between 18° and 25°, are more delicate to be orientated physiologically. The aim of this study was to assess the quality of acetabular orientation by triple pelvic osteotomy (TPO), established by Tönnis and Kalchschmidt, especially with respect to acetabular overcorrection.

**Materials and methods:**

A retrospective examination on 368 consecutive hips treated with TPOs was conducted. On the preoperative pelvic radiograph and the radiographic control 5 days after surgery, LCEA, acetabular index (AI), and anterior (AWI) and posterior wall index (PWI) were measured. According to the above-mentioned definition, the hips were divided into a borderline (*n* = 196) and a dysplastic (*n* = 172) group. Acetabular overcorrection was defined as when LCEA exceeded 35°, AI was below 0° and AWI exceeded 0.60, postoperatively. The postoperative occurrence of a relevant femoroacetabular impingement was correlated to these thresholds. Statistics comprised a priori power analysis, correlation analyses and receiver operating characteristics (ROC).

**Results:**

In the borderline group, in 64 hips (32.7%), LCEA and AI indicated lateral overcorrection. In the dysplastic group, in 14 hips (8.1%), solely AI indicated overcorrection. In none of the hips, relevant anterior overcorrection was detected since AWI never exceeded 0.60. Chi-square test demonstrated a significant correlation between the occurrence of a postoperative femoroacetabular impingement and LCEA exceeding 35°, as well as AI below 0° (*p* < 0.001, resp.). Bravais–Pearson’s analysis showed a significant correlation between the pre- and postoperative values of all parameters in the borderline and the dysplasia group (*p* < 0.001). Thus, ROC analysis could be performed and provided preoperative cutoff values for LCEA (23°) and AI (12.5°), hinting at postoperative overcorrection.

**Conclusion:**

The comparison of radiographic parameters after TPO showed a considerably greater percentage of laterally overcorrected acetabula in the borderline hips than in the dysplastic hips. According to the wall indices, anterior overcorrection was not observed. ROC analysis anticipated unfavorable lateral overcorrection when preoperative LCEA was above 23° and AI below 12.5°. These findings should sensitize the surgeon to the delicate acetabular correction in borderline dysplastic hips.

## Introduction

In modern hip preservation surgery, a differentiated analysis of acetabular orientation is gaining importance. Historically, the lateral center edge angle (LCEA), described by Wiberg as early as in 1939, served as a sole decision criterion in the surgical treatment of developmental dysplasia of the hips (DDH) in adolescents and adults [1]. Based on this parameter, the severity of DDH was classified into “borderline” and “frank”, synonymous with an LCEA between 18° and 25° and below 18°, respectively. However, the literature of the past few years provided robust data confirming the underestimation of acetabular deficiency by LCEA alone, in particular in the borderline range [2–6]. The description of additional parameters, for example, the anterior and posterior wall indices (AWI and PWI) and the acetabular index (AI), allowed a much more comprehensive deformity analysis [7–9]. With the help of the wall indices (AWI and PWI), anterior or posterior under- or overcoverage of the femoral head became objectifiable. In a preliminary examination, a comprehensive deformity analysis was performed on hips labeled borderline dysplastic. The assessment of LCEA, AI, AWI and PWI revealed that around 40% of these hips were deficient either antero-laterally or postero-laterally [10]. The importance of physiological acetabular correction, particularly with regard to the anterior coverage of the femoral head, has been underlined by several examinations. The longevity of the natural hip joint can be compromised by an unfavorable acetabular version: anterior undercorrection will not lead to an unloading of the chondrolabral junction and maintain the symptoms. Even worse, anterior overcorrection will lead to an iatrogenic pincer-type femoro-acetabular impingement, maintain hip pain and promote early-stage osteoarthritis [11–13]. Hartig-Andreasen et al. stated in their work that in mild dysplasia, only little reorientation is possible before overcorrection may occur [14]. This assumption is supported by the day-by-day clinical practice and the impression that acetabula in the borderline range are more delicate to orientate physiologically.

Thus, the aim of this study was to assess to what extent acetabular overcorrection occurs after TPO. We hypothesized that LCEA, AI and AWI might show values according to acetabular overcorrection more often in the borderline cases than in the more dysplastic hips.

## Materials and methods

A retrospective examination on 397 consecutive hips treated with TPOs was conducted. All procedures were performed between January 2015 and December 2021 in our orthopedic department on a total of 324 patients (73 patients treated bilaterally, 276 females, 48 males, mean age 27 years, range 10–48 years). The patients were referred to our hospital mainly with the diagnosis of symptomatic hip dysplasia. Preoperative diagnostic workup included a standardized AP pelvic radiograph. All images were performed in the radiological department of our institution. On these pelvic radiographs, the deformity was analyzed and the correction was planned. The pelvic radiographs were conducted in supine position with a film-focus distance of 1.15 m, the beam centered between the symphysis and a line connecting the anterior superior iliac spines, both legs fully extended and 15° inwardly rotated. Exactly the same standardized technique was used to obtain the pelvic radiographs in the first follow-up examination after TPO, which was scheduled 5 days after the operation. The radiographs were archived in the picture archiving and communication system of our institution (PACS, GE Centricity Universal Viewer Version 6.0, General Electric Healthcare, Chalfort St Giles, UK).

### Classification of the hips into groups

**Fig. 1:**
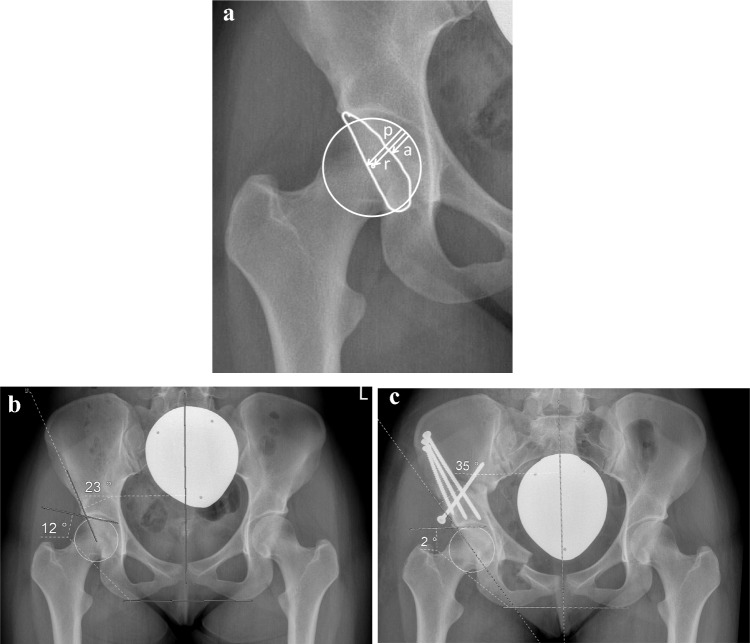
Pelvic radiographs of a 21-year-old female. The patient complained of a load-dependent pain radiating toward the groin area. The physical examination revealed hypermobility with a Beighton score of 9 [21]. A physiotherapeutic approach including specific resistance training for the pelvitrochanteric muscles over a time period of 4 months did not relieve the hip pain. **a** After verification of the usability and the relevant landmarks, first of all, the center of the femoral head was estimated from a circle fit to its contour. For the measurement of the anterior and posterior wall index (AWI, PWI), lines from the medial contour of the circle to its center (radius = *r*), to the anterior wall (short arrow = *a*) and the posterior wall (long arrow = *p*) were drawn. The distances were measured along the femoral neck axis. AWI and PWI were calculated as *a*/*r* and *p*/*r*. In this example, AWI was calculated as 0.45 and PWI 1.05. **b** Then, the longitudinal axis of the pelvis was defined by drawing a vertical line from the processus spinosus of L5 through the middle of the symphysis. The LCEA was measured between the line from the center of the femoral head to the lateral aspect of the sourcil, and the longitudinal axis of the pelvis [1, 22]. Acetabular index was measured between a line connecting the inferior ischial tuberosities and a tangent to the most medial and most lateral aspect of the sourcil. In this example, preoperative LCEA was 23° and AI 12°. **c** Radiograph of the first follow-up 5 days after TPO. Postoperative LCEA of 35° and negative AI of −2° corresponded to a slight lateral overcorrection, as indicated by the preoperative values (see above). With a calculated preoperative cutoff value of 23° for LCEA and 12.5° for AI, ROC analysis anticipated an overcorrection

For this examination, all treated hips were divided in two major groups, based on the LCEA measured on the preoperative pelvic radiograph: (1) borderline hip dysplasia (BHD, “borderline group”), LCEA between 18° and 25° and (2) hip dysplasia (HD, “dysplasia group”), LCEA below 18°. The parameters LCEA, AI, AWI and PWI were measured on all pre- and postoperative pelvic radiographs. The measurement routine is demonstrated in Fig. 1.

### Guideline values for acetabular reorientation and definition of acetabular overcorrection

The TPO was performed in a highly standardized manner, according to the technique described by Tönnis and Kalchschmidt [15]. All procedures were performed by two experienced orthopedic surgeons. This allowed a highly standardized surgical sequence. Intraoperatively, acetabular reorientation was guided by fluoroscopy, which has been proven to be reliable and accurate in a previous study. Because the technical principles of radiography and fluoroscopy differ fundamentally, the c-arm of the fluoroscope has to be adjusted meticulously to match the configuration of the acetabulum from the preoperative radiograph. This is particularly significant for the balancing of the anterior and posterior wall. When this is followed, the radiographic parameters of the acetabulum will show good to excellent correlation between the intraoperative fluoroscopic images and postoperative radiographs [16].

Acetabular orientation pursued the following goals: (1) to achieve a horizontal or slightly upwardly sloping sourcil; (2) to produce an LCEA of 30°; (3) in a posteriorly dysplastic acetabulum: to resolve the crossover sign of the anterior and posterior wall, with the corrected posterior wall running near the center of the femoral head; (4) to avoid anterior overcorrection; (5) in an anteriorly dysplastic acetabulum: to produce sufficient anterior coverage with an estimated AWI around 0.40. The balancing of the anterior and posterior wall was aligned to the normal values of AWI (0.41, range 0.30–0.51) and PWI (0.91, range 0.81–1.14) as described by Siebenrock et al. [7].

For the assessment of lateral and anterior overcorrection, the following thresholds were defined: LCEA > 35°, AI < 0°, AWI > 0.60. In the current literature, there is no coherent definition for LCEA to express acetabular overcoverage. Commonly, the values undulate around 40°. With one of our major objectives to avoid severe acetabular overcorrection, intraoperative fluoroscopy helped us to stay consistently beyond an LCEA of 40° in all cases. In a guideline work by Tannast et al., the authors stated that the upper values of LCEA from large population-based approaches might be falsely high [8]. In their work of radiographic reference values for acetabular under- and overcoverage, the authors defined a mean value of 35° for LCEA regarding overcoverage [8]. For this reason, the threshold of LCEA representing acetabular overcorrection was defined as 35°.

### Clinical examination for femoroacetabular impingement

The strict follow-up regimen 6 weeks, 3 months and 1 year after TPO included a systematic physical examination of the hip. All patient records of the follow-up examinations 3 months and 1 year after surgery were reviewed for a history, indicating femoroacetabular impingement and a positive anterior labral provocation test [flexion, adduction, internal-rotation test (FADDIR)]. With a repeated report on a relevant level of discomfort and a positive anterior labral provocation test, “femoroacetabular impingement” was attributed to the case.

### Exclusion criteria

The following exclusion criteria were applied: (1) radiographs of patients with a severe deformation of the femoral head, e.g., due to Legg–Calve–Perthes disease; (2) radiographs of patients with syndromic diseases.

### Statistical analysis

Intra- and interobserver (observer 1 and 2) correlation of the acetabular parameters measured on the pre- and postoperative radiographs was assessed using intraclass correlation coefficient (ICC). The 95% confidence interval (95% CI) was calculated. The values of ICC were interpreted according to the scale described by Cicchetti: less than 0.40: poor, between 0.40 and 0.60: fair, between 0.60 and 0.75: good, and greater than 0.75: excellent [17]. Correlation between the preoperative and the postoperative parameters (LCEA, AI, AWI, PWI) for the borderline group and the dysplasia group was assessed by Bravais–Pearson’s correlation coefficient, one-tailed, significance threshold *p* < 0.05. High correlation between the parameters was assumed, when the correlation coefficient *r* exceeded 0.5. Correlation between the occurrence of postoperative femoroacetabular impingement (yes/no) and LCEA exceeding 35°, as well as AI below 0° was expressed by Pearson’s Chi-square test. Analysis of preoperative cutoff values for postoperative overcorrection was performed with receiver operating characteristics (ROC). The statistical analysis and presentation were performed using SPSS Statistics, Version 26 (IBM, Armonk, New York, USA).

A priori power analysis indicated a minimum sample size of 84 cases for ICC, two-tailed and of 67 cases for Pearson’s correlation, one-tailed (power 0.80, α set to 0.05, respectively) (G*Power Version 3.1.9.6).

## Results

According to the LCEA, grouping of the 397 treated hips resulted in 201 hips in the borderline range (BHD, LCEA between 18° and 25°) and 196 hips with a frank hip dysplasia (HD, LCEA below 18°). After application of the exclusion criteria, 368 hips were suitable for further examination: 196 hips (53.3%) were included in the borderline group and 172 (46.3%) hips in the dysplasia group. In the borderline group, three hips were excluded due to a severe deformation of the femoral head after Legg–Calve–Perthes disease, and two hips were excluded due to a syndromic disease. In the dysplasia group, 10 hips were excluded due to a deformation of the femoral head (e.g., coxa magna after Legg–Calve–Perthes disease or asphericity in severe hip dysplasia) and 14 hips due to a syndromic disease, respectively. Finally, the borderline group showed a gender distribution of 183 female and 13 male hips (6.6% male hips), and the dysplasia group 148 female and 24 male hips (14.0%), respectively. For the sake of clarity, the results of the pre- and postoperative measurements and the demographics are displayed in Tables 1 and 2.

**Table 1:** Results of all measured parameters of the hips in the borderline and the dysplasia groups, mean values and standard deviation (SD)

	Preoperative measurements (mean, SD)				Postoperative measurements (mean, SD)				Δ pre- to postoperative measurements (mean)			
	LCEA	AI	AWI	PWI	LCEA	AI	AWI	PWI	LCEA	AI	AWI	PWI
Total	15.9° (± 7.2°)	15.6° (± 7.6°)	0.35 (± 0.12)	0.85 (± 0.15)	28.1° (± 4.5)	3.3° (± 5.2°)	0.35 (± 0.09)	0.99 (± 0.13)	+ 12.2°	−12.3°	± 0	+ 0.14
Boderline dysplasia	20.9° (± 2.1°)	11.2° (± 4.2°)	0.39 (± 0.11)	0.88 (± 0.13)	30.4° (± 3.0°)	0.9° (± 3.8°)	0.36 (± 0.08)	1.01 (± 0.10)	+ 9.5°	−10.3°	−0.03	+ 0.13
Dysplasia	10.3° (± 6.7°)	20.6° (± 7.4°)	0.31 (± 0.11)	0.82 (± 0.16)	25.4° (± 4.4°)	5.9° (± 5.4°)	0.33 (± 0.09)	0.97 (± 0.15)	+ 15.1°	−14.7°	+ 0.02	+ 0.15

The columns on the right display the differences between the pre- and the postoperative values

**Table 2:** Demographics of the study groups

	*n*	Gender distribution (female: male) (*n*) (percent of total)	Age in years at surgery (mean, range)	Right: left hip (*n*) (percent of total)
Included total	368	331:37 (89.9%:10.1%)	27.4 (10–48)	221:147 (60.1%:39.9%)
Borderline group	196	183:13 (93.4%:6.6%)	27.6 (14–46)	106:90 (54.1%:45.9%)
Dysplasia group	172	148:24 (86.0%:14.0%)	27.2 (10–48)	115:57 (66.9%:33.1%)

Interestingly, in the dysplasia group, two-thirds of the symptomatic hips needing TPO were right hips. We have no conclusive explanation for this unexpected distribution

Inter- and intraobserver correlation analysis showed throughout excellent values according to Cicchetti: the lowest values were calculated for the interrater reading of AWI on the postoperative radiographs (0.795, 95% CI 0.685–0.867), and the highest values for the intraobserver reading of AI on the preoperative radiographs (0.988, 95% CI 0.978–0.990) [17].

Bravais–Pearson’s analysis showed a significant correlation between the pre- and postoperative values of all parameters in the borderline and the dysplasia group (*p* < 0.001). For LCEA, the correlation coefficient resulted in *r* = 0.411 (borderline group) and *r* = 0.637 (dysplasia group), for AI: *r* = 0.614 and *r* = 0.729, for AWI: *r* = 0.373 and *r* = −0.232, and for PWI: *r* = 0.539 and *r* = 0.604, respectively.

Person’s Chi-square test demonstrated a significant correlation between the occurrence of a postoperative femoroacetabular impingement and LCEA exceeding 35° (*p* < 0.001), as well as AI below 0° (*p* < 0.001) (total of 28 hips, 5 originating from the dysplasia group, 23 from the borderline group, resp.).

**Fig. 2:**
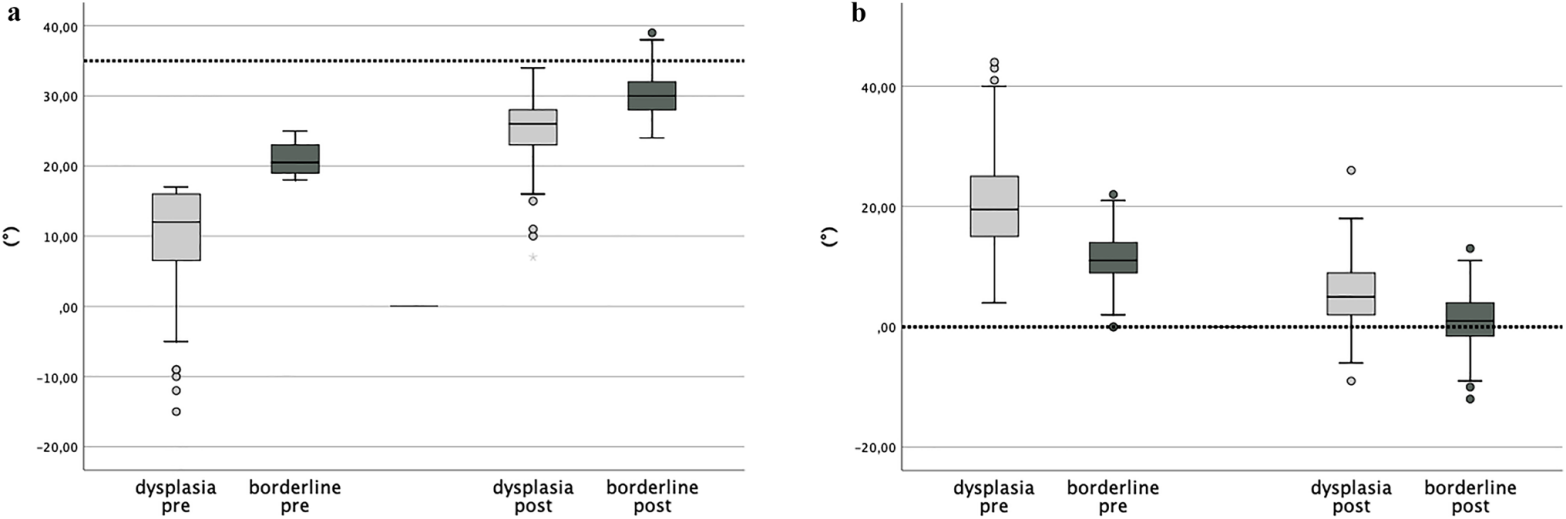
**a** The box plots (representing from top to bottom: maximum, first quartile, median, third quartile and minimum) show the pre- and postoperative values for LCEA, dysplasia group light gray, and borderline group dark gray. By definition, all preoperative values for the borderline group range between 18° and 25°. The dotted bar at 35° represents the threshold to an unfavorable overcorrection. The boxplot on the very right shows that some postoperative values exceeded the defined threshold for overcorrection. **b** The box plots show the pre- and postoperative values for AI, dysplasia group light gray and borderline group dark gray. The dotted bar represents the threshold to an overcorrection. Both box plots on the right side show that only a few postoperative values from the dysplasia group and several values from the borderline group were below the threshold

**Fig. 3:**
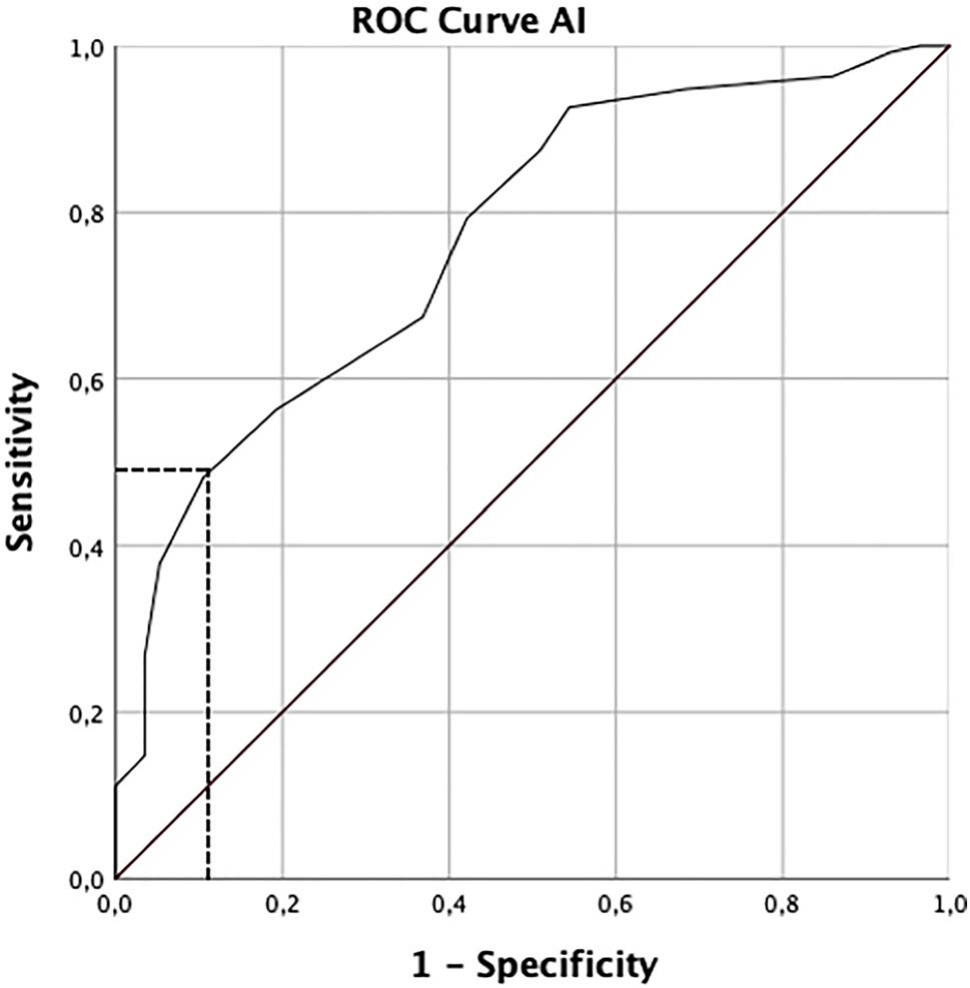
Receiver operating characteristics (ROC) curve, using the example of the parameter AI. The intersection point on the curve (sensitivity of 0.5 and a specificity of 1–0.1) marks the best possible compromise between sensitivity and specificity to anticipate an unfavorable postoperative AI below 0° (downwardly sloping sourcil). This corresponded to a preoperative cutoff value of 12.5° for AI

According to the above defined criteria, in 78 from 368 hips (21.2%) the postoperative measurements indicated acetabular overcorrection. Of these, 14 hips originated from the dysplasia group (8.1%), showing AI < 0° as the only pathological parameter. The other 64 hips originated from the borderline group (32.7%), in 21 LCEA exceeded 35°, in 57 AI was measured to be less than 0°, and in 14 hips LCEA and AI both were beyond the defined thresholds (Fig. 2). AWI never exceeded 0.60, synonymous with the absence of a relevant anterior overcorrection. With the prerequisite of a significant correlation between the pre- and postoperative values, ROC analysis was performed for the parameters LCEA and AI and provided preoperative cutoff values for an unfavorable postoperative overcorrection: LCEA: 23° (postoperative state variable: > 35° = 1, < 35° = 0) and AI: 12.5° (> 0° = 1, < 0° = 0) (Fig. 3).

## Discussion

The most important finding in this examination was that the remarkable difference of the percentage of acetabular overcorrection between the dysplasia and the borderline group (8.1% vs 32.7%). In the dysplasia group, AI solely measured up to a slightly downwardly sloping sourcil, whereas in the borderline group, in several cases, additionally LCEA above 35° indicated a more pronounced overcorrection.

As early as 1999, Tönnis and Heinecke reported on the problem of excessive anterior acetabular coverage, along with acetabular retroversion. The pathomorphology was described as a risk factor for premature hip joint degeneration [18]. Measurement methods to objectify anterior and posterior coverage of the femoral head, such as the AWI and PWI, had not been established at that time. In 2008, Tönnis, as one of the protagonists of TPO, strongly recommended avoiding anterior overcorrection of the acetabulum, since this was recognized as a promotor for ongoing osteoarthritis [19]. In recent years, increasing knowledge about the implications of unfavorable acetabular orientation emerged. Nowadays, overlooking approximately three decades of TPO and periacetabular osteotomy (PAO), the understanding of physiological acetabular orientation is more comprehensive. Risk factors such as excessive anterior acetabular overcorrection or acetabular undercorrection have been described quite well and confirmed the rather descriptive findings of Tönnis and Heinecke [7, 8, 11–13]. Based on these findings, objectifiable acetabular parameters have been established, guiding the surgeon through the deformity analysis as well as the planning and the execution of acetabular correction.

Nevertheless, although guideline values for the acetabular correction (see above) were available, the measured parameters indicated lateral overcorrection in several of our patients. Our measurements revealed a mean reduction of the LCEA and AI of approximately 15° in the dysplastic group and 10° in the borderline group. This shows that a differentiated correction was possible, taking into account the severity of the dysplasia. Regarding the mean values for the postoperative correction, a large proportion of the acetabula hips in the borderline group were corrected toward the target figures (see above, Fig. 2). But compared to the dysplasia group, a larger percentage of the hips in the borderline group were overcorrected laterally, meaning that LCEA exceeded 35° and AI was below 0°. It has to be mentioned that in the 196 hips of the borderline group, the greatest discrepancy from the target values in a single outlier was measured with 39° for LCEA and −12° for AI. In none of the hips AWI exceeded 0.59, so relevant anterior overcorrection did not occur.

Our findings underline the results of Hartig-Andreasen et al. The authors examined radiographic predictors for hip arthroscopy 2 years after POA. It was stated that in “mild dysplasia” with an LCEA of 20°–25°, only little reorientation is possible before overcorrection may occur. The authors assumed that this could be the reason for the finding of an LCEA of 20°–25° being a significant predictor for subsequent arthroscopy. Interestingly, a negative AI was not a significant factor [14].

In the present examination, particularly in the borderline group, the postoperative values of LCEA and AI indicated a relevant percentage of overcorrected acetabula. Since the pre- and postoperative values for the parameters LCEA and AI correlated significantly, ROC analysis was performed. This provided preoperative cutoff values with 23° for LCEA and 12.5° for AI which might help to sensitize the surgeon to imminent overcorrection. The impact of overcorrection is underlined by the clinical data of this examination. The occurrence of iatrogenic femoroacetabular pincer-type impingement correlated significantly with an LCEA exceeding 35° and an AI below 0° after TPO. Hartig-Andreasen et al. described that 27% of their patients underwent hip arthroscopy within a 2-year follow-up after PAO, predominantly to perform trimming of the anterior acetabular rim and labral reinsertion.

In our patient records of the follow-up at 3 months and 1 year postoperatively, 28 cases (of 368 cases, 7.6%) with persistent symptoms according to a femoroacetabular impingement were found. Cases with symptoms according to a persistent overloading of the chondrolabral junction (hip-related pain in upright activities), potentially due to a persistent microinstability of the joint, presenting a positive anterior labral provocation test in addition to a positive flexion, abduction and external rotation test (FABER), were not labeled “femoroacetabular impingement”. In 11 (of 368 cases, 3%) cases with femoroacetabular impingement, hip arthroscopy was performed within the first 2 years after TPO. In almost all cases, hip arthroscopy was performed simultaneously with the removal of the osteosynthesis implants. With a remarkably high number of hip arthroscopies performed after PAO, it has to be stressed out that the results of Hartig-Andreasen et al. cannot be compared to the present examination, since they indicated hip arthroscopy due to more pathologies than femoroacetabular impingement [14].

Interestingly, in the borderline group, the male hips made up less than half of the percentage than in the dysplasia group. This remarkable difference might reflect that males are able to compensate better for a slightly reduced coverage of the femoral head. Presumably, males might experience less microinstability due to less ligamentous laxity and more centering pelvitrochanteric muscle force, compared to their female fellow patients [20]. This specific issue has to be subject to further examination.

This examination has the following limitations. First, the two observed groups showed differences in the gender distribution. Since it is known that male dysplastic acetabula tend to be slightly more deficient postero-laterally than female, this might have had an influence on the results. Second, hips of patients with a syndromic disease or a severe deformation of the femoral head (e.g., due to Legg–Calve–Perthes disease) were excluded. For this reason, the results of this examination cannot be transferred to these hip conditions. Third, this examination did not integrate patient-related outcome scores, so the clinical relevance of the observed overcorrection has to be assessed in an upcoming study.

## Conclusion

The comparison of radiographic parameters after TPO showed a greater percentage of laterally overcorrected acetabula in the borderline hips than in the dysplastic hips. The postoperative wall indices did not show anterior overcorrection, in the borderline hips or the dysplastic hips. ROC analysis indicated unfavorable lateral overcorrection when preoperative LCEA was above 23° and AI below 12.5°. When TPO is considered in the treatment of borderline dysplastic hips, the surgeon should be sensitized to the rather delicate acetabular correction. The scientific evaluation whether these findings are associated with clinical results will be of particular future interest.

## Data Availability

The authors agree to deposit the data that support the findings of this examination. The data have not been uploaded to a public repository yet.
